# Prognostic Impact of the Early Use of Tolvaptan in Elderly Patients with Acute Decompensated Heart Failure

**DOI:** 10.3390/jcm12093105

**Published:** 2023-04-24

**Authors:** Tomoaki Okada, Toru Miyoshi, Akihiro Oka, Ryu Tsushima, Yuya Sudo, Kosuke Seiyama, Wataru Takagi, Tomohiro Kawaguchi, Masatomo Ozaki, Masahiro Sogo, Satoko Ugawa, Kazumasa Nosaka, Masahiko Takahashi, Keisuke Okawa, Masayuki Doi

**Affiliations:** 1 Department of Cardiology, Kagawa Prefectural Central Hospital, 1-2-1 Asahi-machi, Takamatsu 760-8557, Japan; 2 Department of Cardiovascular Medicine, Okayama University Graduate School of Medicine, Dentistry and Pharmaceutical Sciences, 2-5-1 Shikata-cho, Okayama 700-8558, Japan

**Keywords:** acute decompensated heart failure, elderly patients, prognosis, tolvaptan

## Abstract

The number of elderly patients with acute decompensated heart failure (ADHF) is increasing, and it is often difficult to treat. This study aimed to evaluate the efficacy and safety of using tolvaptan early after hospitalization in elderly patients with ADHF and the prognosis one year after hospitalization. This study enrolled 185 patients with ADHF who were admitted for the first time. Tolvaptan was administered within 24 h after admission. These patients were assigned to two groups: over 80 years old (*n* = 109) and under 80 years old (*n* = 76). There were no significant differences between the two groups in the occurrence of MACCE within one year (25% vs. 20%, *p* = 0.59). All-cause mortality was significantly higher in the over-80 group (12% vs. 2%, *p* = 0.01). There were no significant differences in the incidence of worsening renal failure (11% vs. 7%, *p* = 0.46) and hypernatremia (5% vs. 9%, *p* = 1.0), and in the duration of hospitalization (19.2 days vs. 18.8 days, *p* = 0.8). Tolvaptan might be effective and safe in elderly patients with ADHF, and there was no difference in the incidence of MACCE within one year.

## 1. Introduction

In recent years, the incidence of heart failure (HF) has increased in developed countries, posing a public health concern [1,2]. As life expectancy grows, the proportion of elderly patients with HF has been increasing. Elderly patients with HF often require a longer duration of hospitalization and often have a poor prognosis [3]. Furthermore, a nationwide registry reported that hospitalized patients with acute decompensated HF (ADHF) in Japan were elderly, with a poor prognosis [4]. Age is a significant and independent predictor of in-hospital and long-term mortality in patients with HF [2]. Elderly patients with HF have a high rate of comorbidities, such as anemia, chronic kidney disease (CKD), and chronic obstructive pulmonary disease (COPD), and may be resistant to treatment [5,6,7].

Tolvaptan (TLV), an oral vasopressin-2 receptor antagonist, acts on the distal nephron, reducing volume overload and improving congestive symptoms associated with HF through potent diuresis [8]. TLV was administered during the acute phase of hospitalization in patients with HF in the Endovascular Valve Edge-to-Edge Repair Study (EVEREST) trial, and it improved symptoms and reduced body weight [9]. In recent years, TLV has been used as one of the therapeutic agents for HF patients with volume overload along with loop diuretics in Japan. The efficacy and safety of TLV in elderly patients with HF have been reported in the Surveillance In Heart faiLurE (SMILE) study and other previous studies [10,11,12,13]. However, few studies have investigated the outcomes of elderly patients treated with TLV during the hyperacute phase of HF. Therefore, this study aimed to re-evaluate the efficacy and safety of using TLV earlier in patients with HF, aged 80 years or older requiring hospitalization, and to evaluate the prognosis within one year.

## 2. Materials and Methods

### 2.1. Patients

This was a single-center, retrospective, observational study. We enrolled 592 patients with ADHF admitted to the Kagawa Prefectural Central Hospital between April 2017 and March 2020. Symptoms and signs of HF were defined according to the Framingham criteria [14]. ADHF was defined as either new-onset HF or decompensation of chronic heart failure with symptoms sufficient enough to warrant hospitalization, resulting in the need for urgent therapy. The participants in this study were newly hospitalized patients with ADHF. After the diagnosis of HF, initial treatment was performed according to the physician’s discretion when the patients were admitted to our hospital. TLV was administered within 24 h of admission when possible if the inclusion criteria of systemic congestion and absence of hypernatremia (serum sodium levels ≥ 145 mEq/L) were met.

**Figure 1:**
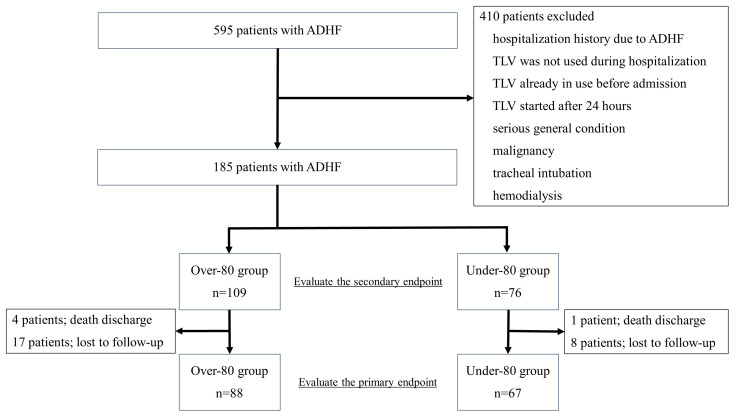
Study flow chart. Overall, 595 patients with ADHF were administered TLV in our hospital during the study period; 410 patients were excluded owing to the exclusion criteria, and 185 patients were enrolled. ADHF, acute decompensated heart failure; *n*, number; TLV, tolvaptan.

Overall, 410 patients were excluded based on the following exclusion criteria: previous hospitalization for ADHF, TLV not used, TLV already in use, TLV started after 24 h, serious general condition, malignancy, tracheal intubation, or hemodialysis. Thus, 185 patients were enrolled and evaluated (Figure 1). Patients with eGFR < 30 mL/min/m^2^ were also included because renal dysfunction was not included as an exclusion criterion. Some of these included patients were already on any kind of antihypertensive, antiarrhythmic or antidiabetic medications.

The patients were assigned to two groups based on their age: the over-80 group (age ≥ 80 years, mean 85 ± 4.3 years, *n* = 109) and the under-80 group (age < 80 years, mean 64 ± 11.2 years, *n* = 76). This study was approved by the ethics committee of the Kagawa Prefectural Central Hospital. The requirement for informed consent was waived because of the low-risk nature of the study and the inability to obtain consent directly from all study participants. Instead, we publicized the study protocol extensively at the Kagawa Prefectural Central Hospital and on the hospital website (http://www.chp-kagawa.jp/, accessed on 29 September 2021) and provided patients with the opportunity to withdraw from the study. This study was conducted in accordance with the principles of the Declaration of Helsinki.

### 2.2. Use of TLV and Conventional Primary Therapy

All included patients were administered TLV within 24 h of admission, when possible. Based on the treating physician’s judgment, the initial dose of TLV (15 mg, 7.5 mg, or 3.75 mg once daily) was determined with reference to the patient’s background, such as age, body weight, blood pressure, severity of HF, and renal function. Dosing, number of days of administration, and the decision to continue or discontinue TLV after HF improvement were left to the physician’s discretion.

The conventional primary therapy for ADHF was based on the physician’s judgment, such as oxygen inhalation with a mask; adjustment of the sitting position; or administering diuretics (intravenous or oral), vasodilators, or inotropes; and noninvasive positive pressure ventilation (NPPV). All patients were treated with optimal medical therapy for HF during in-hospital periods, including diuretics, beta blockers, aldosterone antagonists, angiotensin-converting enzyme inhibitors, and angiotensin II receptor blockers.

### 2.3. Study Endpoints

The primary endpoint of this study was the incidence of major adverse cardiac and cerebrovascular events (MACCE), including all-cause mortality and rehospitalization within one year. These endpoints were assessed regardless of the continual use of TLV at discharge. We considered the incidence of all-cause death, cardiac death, HF death, noncardiac death, rehospitalization due to HF, cerebral infarction, and fatal arrhythmia as MACCE.

The secondary endpoints of this study were the efficacy and safety of TLV and in-hospital mortality during hospitalization. The efficacy outcomes of TLV were assessed based on the length of hospital stay, total urine volume, and mean change in body weight. The total urine volume was assessed 24 and 48 h after TLV administration. The mean change in body weight was assessed at 7 days and at discharge. The safety outcomes of TLV were assessed based on hypernatremia and worsening renal failure (WRF). Hypernatremia was defined as an increase in serum sodium levels of ≥150 mEq/L within three days of admission and in-hospital periods. WRF was defined as an absolute increase in serum creatinine of 0.3 mg/dL or 50% from baseline within three days of admission.

### 2.4. Statistical Analysis

Continuous variables are expressed as mean ± standard deviation, while categorical variables are expressed as numbers (proportions). Student’s *t*-test was used to compare continuous variables in the baseline characteristics. Based on the sample size, categorical data were compared using chi-square analysis and the Fisher’s test. Efficacy analysis was performed according to the treatment received, based on an intention-to-treat analysis. All statistical analyses were performed using IBM SPSS version 24 (IBM, Armonk, NY, USA). Statistical significance was set at *p* < 0.05.

## 3. Results

The baseline characteristics of the patients are presented in Table 1.

There were several differences in patients’ background between the two groups. The patients’ mean age was 85 and 64 years in the over-80 and under-80 groups, respectively. The over-80 group had a lower body weight and body mass index (BMI), and a higher proportion of CKD and atrial fibrillation (AF), than the under-80 group. Ischemic heart disease, hypertensive heart disease, and cardiomyopathy, including dilated cardiomyopathy, were not significantly different between the two groups. In the under-80 group, the left ventricular ejection fraction (LVEF) tended to decrease, and the left ventricle tended to dilate. Laboratory data showed no difference between the two groups in total protein, albumin, and brain natriuretic peptide levels.

In contrast, hemoglobin, estimated glomerular filtration rate (eGFR), and total cholesterol levels were lower in the over-80 group. The use of carperitide, inotropes, and NPPV after admission did not differ between the two groups. TLV was administered in both groups shortly after admission, and the initial dose was higher in the under-80 group. Table 2 presents laboratory data and medications used at discharge. LVEF, left ventricular end-diastolic diameter; hemoglobin level; and eGFR showed the same trends as at the time of admission.

Table 2 shows the laboratory data and medications at discharge. At discharge, 60% of the patients in the over-80 group and 57% in the under-80 group continued TLV use, with no significant difference between the two groups. There was no difference in the TLV dose between the two groups.

### 3.1. Primary Endpoints

**Figure 2:**
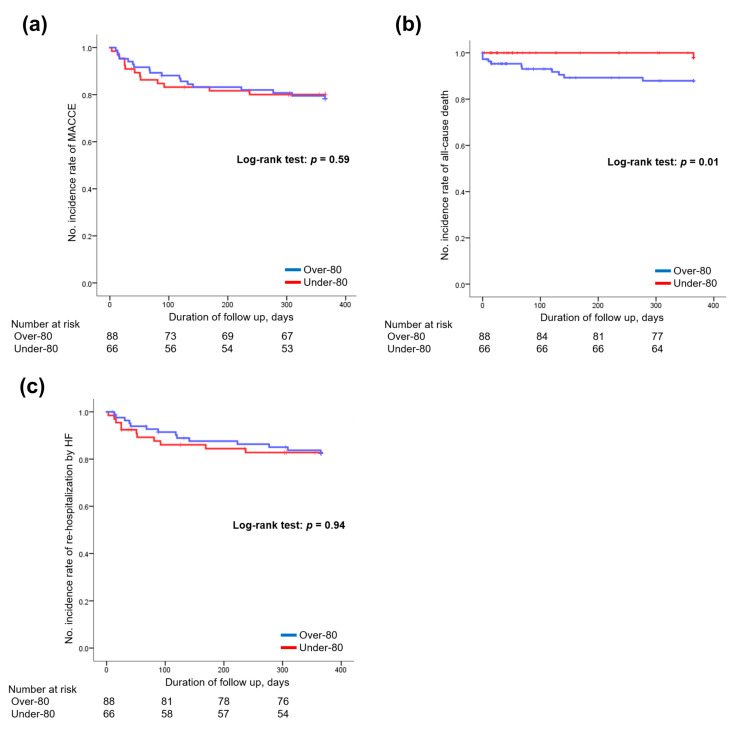
Clinical events within 1 year of discharge. There was no significant difference in the incidence of MACCE (**a**) or rehospitalization owing to HF (**c**). All-cause mortality was significantly higher in the over-80 group than that in the under-80 group (*p* = 0.01; (**b**)). MACCE, major adverse cardiac and cerebrovascular events; HF, heart failure.

Table 3 and Figure 2 show the results for the primary endpoints.

In the over-80 group, four patients died during hospitalization, and 17 patients were lost to follow-up; in the under-80 group, one patient died during hospitalization, and eight patients were lost to follow-up. Thus, 88 patients in the over-80 group and 67 patients in the under-80 group were evaluated. At one year, the follow-up rate was 80% in the over-80 group and 88% in the under-80 group (*p* = 0.22).

The median observation period was 365 (76–365) days in the over-80 group and 365 (89–365) days in the under-80 group. There was no significant difference between the two groups in the incidence of MACCE within one year (25% in the over-80 group vs. 20% in the under-80 group, *p* = 0.59; Figure 2a). All-cause mortality was significantly higher in the over-80 group than in the under-80 group (12% in the over-80 group vs. 2% in the under-80 group, *p* = 0.01; Figure 2b). Conversely, there was no difference in rehospitalization due to HF within one year between the two groups (18% in the over-80 group vs. 17% in the under-80 group, *p* = 0.94; Figure 2c).

### 3.2. Secondary Endpoints

**Figure 3:**
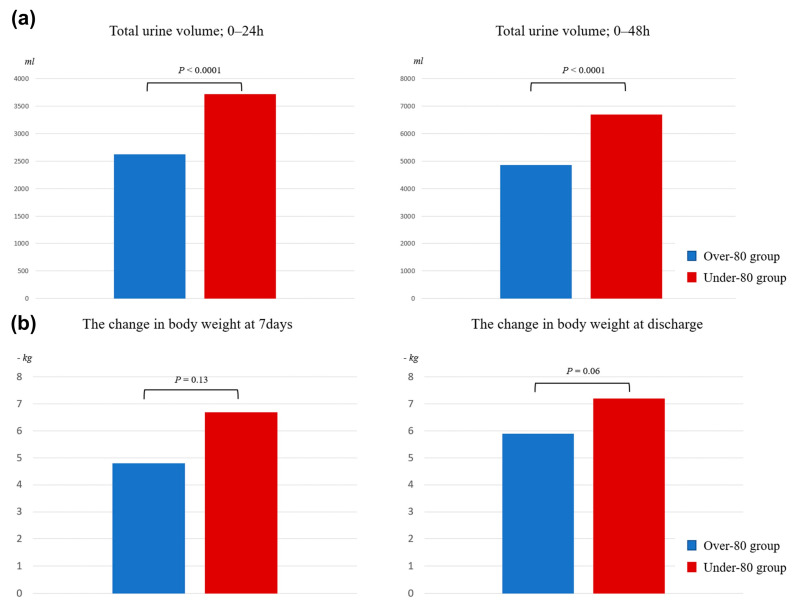
Total urine volume and the change in body weight during hospitalization periods. (**a**) The total urine volume at 24 and 48 h; (**b**) The change in body weight at 7 days and at discharge.

Table 4 and Figure 3 show the results for the secondary endpoints.

The incidence of in-hospital death was observed in four patients in the over-80 group and one patient in the under-80 group. The total urine volume at 24 h and 48 h was 2620 ± 1227 mL and 4854 ± 1969 mL in the over-80 group and 3725 ± 2244 mL and 6695 ± 3951 mL in the under-80 group, respectively (*p* ≤ 0.0001 at 24 h, *p* ≤ 0.0001 at 48 h; Figure 3a).

The mean change in body weight at discharge was −5.9 ± 3.5 and −7.2 ± 4.8 in the over-80 and under-80 groups, respectively (*p* = 0.06; Figure 3b). The total urine volume was significantly higher in the under-80 group; however, weight loss was similar in both groups, and there was no difference in the length of hospitalization between the two groups. The incidences of hypernatremia and WRF did not differ between the two groups.

## 4. Discussion

In this study, the safety and efficacy of TLV for elderly patients with HF were similar to those reported previously [11,12]. However, our study differs from previous studies in that it investigated cardiovascular events over a period of one year in patients with ADHF who were administered TLV immediately after hospitalization. The main findings were as follows: First, there were no significant differences between the two groups in the occurrence of MACCE within one year. Second, despite an unfavorable background in the over-80 group, the incidence of WRF and hypernatremia did not differ significantly compared to the under-80 group, and the body weight decrease was also similar. Third, the incidence of in-hospital death and length of hospitalization did not differ between the two groups.

Previous studies reported the efficacy and safety of TLV administration within 24 h in elderly patients with ADHF [11,15]. Our study also demonstrated the efficacy of the early administration of TLV within 24 h after admission in patients with ADHF, aged over-80 years, and in patients aged under-80 years. One of the effects of early administration of TLV is thought to be a shortened length of hospital stay. Matsukawa et al. [16] reported that the early use of TLV shortened the length of hospital stay in patients with HF aged 75 years or older. Elderly patients with ADHF are likely to experience a decline in the activities of daily living owing to long-term bed rest, leading to difficulties in living after discharge. Early use of TLV and shorter lengths of hospital stay may be effective in maintaining the quality of life after hospital discharge.

Elderly patients with HF have complex medical conditions and may be challenging to treat. Previous studies have reported that elderly patients with HF often have CKD, anemia, AF, and COPD [6,7]. In our study, more patients with anemia, AF, CKD, and low BMI were observed in the over-80 group, suggesting that the patient background is more complicated in the elderly. In recent years, the efficacy and safety of TLV have also been reported in very elderly patients with HF aged 90 years or older [12,13]. Our study revealed the efficacy of TLV in first-time hospitalizations for ADHF; however, the efficacy of the early administration of TLV in elderly patients after repeat hospitalization for ADHF has not been reported [17]. TLV efficacy in elderly patients with HF with more complex medical conditions may be established in future studies.

A unique point of our study was that we investigated the outcomes one year after hospital discharge in patients with ADHF treated with the early administration of TLV. All-cause mortality was higher in the over-80 group, but there was no difference between the two groups in the occurrence of MACCE or rehospitalization for heart failure. ADHF in the elderly is an increasingly common clinical problem, with high mortality rates worldwide [18]. The number of elderly patients with ADHF is only expected to increase in Japan [19]. Furthermore, a nationwide registry reported that hospitalized patients with ADHF in Japan were very elderly, with a poor prognosis [4]. In this Japanese registry of ADHF, the one-year all-cause death was reported to be 14.2% and the one-year hospitalization for HF was 29.4%. This result was higher than our finding, especially in HF rehospitalization, even in the over-80 group. Therefore, it is necessary to further investigate the long-term effects of the early administration of TLV in the acute phase of ADHF.

Regarding TLV use in the acute phase of ADHF, previous studies, including ours, have shown the efficacy of TLV not only within 24 h but also early after admission [11,12,20]. In addition, TLV has been reported to be effective in shortening the length of hospitalization in patients with ADHF requiring intensive care [21]. It has also been reported that lower doses of TLV may be safer in elderly patients with heart failure [10]. Even after discharge, the long-term use of TLV has been reported to delay rehospitalization for HF in patients with severe HF [22]. Furthermore, it has been suggested that the use of long-term low-dose TLV (<7.5 mg/day) may be favorable for reducing HF rehospitalization [23].

Pulmonary and systemic congestion are important factors in patients with ADHF and are present in many patients at the time of hospitalization [24]. Various effects associated with continued congestion have been identified, and residual congestion is known to be an important factor that can worsen prognosis and increase rehospitalization [25]. TLV has been used for decongestion, but the appropriate use of TLV has not yet been established.

In recent years, the drugs for treating HF have changed significantly. In patients with HF with reduced ejection fraction, the estimated aggregate benefit is maximal for a combination of angiotensin receptor–neprilysin inhibitors, beta blockers, mineralocorticoid receptor antagonists, and sodium–glucose cotransporter-2 inhibitors [26]. In addition, early decongestion is important because the relationship between residual congestion and prognosis has been reported in the acute phase treatment of heart failure [27]. Loop diuretics, such as furosemide, remain the mainstream therapy and are often used to reduce fluid retention in patients with ADHF. On the other hand, diuretic resistance is often a problem [28]. TLV is an extremely effective drug for decongestion; however, the impact of rapid decongestion by the early use of TLV in patients with ADHF on the event occurrence of MACCE within one year is still unclear. It is necessary to investigate its optimal usage in the future.

The optimal use of TLV requires safety considerations. It is important to avoid causing renal dysfunction or electrolyte abnormalities. Elderly patients with HF frequently have CKD. Complications of WRF during ADHF treatment prolong the hospitalization period and increase the HF rehospitalization rate and mortality after one year [29,30]. In addition, previous studies have reported that hypernatremia was associated with a high mortality rate in the intensive care unit [31,32]. In our study, there was no difference in the occurrence of adverse events such as hypernatremia and WRF between the over-80 and under-80 groups during their hospital stay. On the other hand, the effect of TLV on renal function after discharge could not be evaluated, and further studies are needed in the future. Previous studies have also reported that HF treatment using TLV was performed in elderly patients with ADHF without causing adverse events such as WRF or hypernatremia [11,12,13]. These findings revealed that elderly patients can use TLV without adverse events and achieve clinical effectiveness.

This study had some limitations. First, this was a single-center, retrospective, observational study with a small sample size. Second, this study included only hospitalized patients with ADHF who used TLV for the first time. Third, the administration of TLV, initial dose of TLV, administration periods of TLV, and other initial therapies were decided based on the physician’s judgment. Finally, we could not investigate the continuation and dose of TLV after discharge and did not evaluate the long-term effects of TLV. Therefore, we could not evaluate the direct effects of TLV on acute treatment during hospitalization or postdischarge effects, in patients with ADHF. It is unclear whether early and short use of TLV affects the occurrence of MACCE within one year. In addition, long-term prognosis may be affected by interventions other than TLV administration for heart failure, and comorbidities other than heart disease. However, this study showed the real-world prognosis of patients with ADHF who received acute-phase treatment using TLV and drug adjustments, including diuretics, depending on their condition. A randomized, multicenter, large-scale trial is required to confirm the direct effect of TLV.

## 5. Conclusions

This study showed the usefulness of the early use of TLV in patients with ADHF on MACCE within one year. The over-80 group had the same outcomes as the under-80 group, and there was no difference in the occurrence of WRF and hypernatremia in the acute phase of HF and in MACCE within one year. All-cause mortality, however, was significantly higher in the over-80 group.

## Figures and Tables

**Table 1:** Baseline characteristics.

	Over-80	Under-80	
Characteristics	*n* = 109	*n* = 76	*p*-Value
Age, years	85 ± 4.3	64 ± 11.2	<0.0001
Men	58 (53)	47 (61)	0.29
Body weight, kg	56.6 ± 11.3	69.1 ± 21.6	<0.0001
Body mass index, kg/m^2^	23.6 ± 3.7	26.2 ± 6.1	0.001
Systolic blood pressure, mmHg	150.9 ± 36.7	145.2 ± 37.9	0.31
Diastolic blood pressure, mmHg	81.7 ± 24.6	91.6 ± 24.7	0.009
Pulse rate, bpm	87.3 ± 25.9	103.5 ± 28.0	0.0001
NYHA Ⅲ to Ⅳ	98 (89)	72 (94)	0.28
Clinical scenario (CS)			
CS1	41 (37)	32 (42)	0.54
CS2	65 (59)	40 (52)	0.36
CS3	3 (2)	4 (5)	0.44
Medical history			
Hypertension	82 (75)	49 (64)	0.13
Diabetes mellitus	37 (33)	21 (27)	0.42
Dyslipidemia	32 (29)	25 (32)	0.63
Chronic kidney disease	59 (54)	23 (30)	0.001
Cerebral infarction	11 (10)	9 (11)	0.81
History of smoking	47 (43)	45 (59)	0.03
Etiology of heart failure			
Ischemic heart disease	33 (30)	21 (27)	0.74
Hypertensive heart disease	56 (51)	37 (48)	0.76
Atrial fibrillation	59 (54)	26 (34)	0.01
Valvular disease	30 (27)	10 (13)	0.02
Cardiomyopathy	9 (8)	12 (15)	0.15
Medications prior to admission			
ACEi/ARB	36 (33)	14 (18)	0.02
Beta blocker	37 (33)	14 (18)	0.02
Aldosterone blocker	17 (15)	7 (9)	0.26
Loop diuretics	42 (38)	19 (25)	0.05
Laboratory data at admission			
LVDd, mm	49.2 ± 7.0	56.0 ± 9.4	0.0001
LVEF, %	48.6 ± 15.3	34.9 ± 16.3	0.0001
Hemoglobin, g/dL	11.0 ± 2.1	12.9 ± 5.1	<0.0001
Total protein, g/dL	6.4 ± 0.7	6.3 ± 0.7	0.2
Albumin, g/dL	3.3 ± 0.4	3.4 ± 0.5	0.19
BUN, mg/dL	27.5 ± 12.0	22.2 ± 12.4	0.004
Creatinine, mg/dL	1.3 ± 0.6	1.2 ± 0.7	0.29
eGFR, mL/min/1.73 m^2^	42.7 ± 18.7	57.0 ± 28.6	0.0002
Serum Na, mEq/L	139.0 ± 9.8	140.5 ± 4.2	0.18
Serum K, mEq/L	4.2 ± 0.7	4.1 ± 0.6	0.39
Total cholesterol, mg/dL	144.7 ± 31.5	165.4 ± 46.4	0.001
EPA/AA	0.28 ± 0.19	0.23 ± 0.17	0.14
BNP, pg/mL	1211.2 ± 1708	1089 ± 914	0.53
Medications at admission			
Carperitide	53 (48)	34 (44)	0.65
Inotropes	10 (9)	7 (9)	1
NPPV	25 (22)	14 (18)	0.58
Administration of tolvaptan			
Day of initiation, days	1.2 ± 0.8	1.2 ± 0.6	0.62
Initial dose, mg	9.6 ± 3.6	10.8 ± 3.7	0.04

Values are presented as the mean ± standard deviation or *n* (%). AA, arachidonic acid; ACEi, angiotensin-converting enzyme inhibitor; ARB, angiotensin receptor blocker; BNP, brain natriuretic peptide; BUN, blood urea nitrogen; bpm, beat per minute; CS, clinical scenario; eGFR, estimated glomerular filtration rate; EPA, eicosapentaenoic acid; LVDd, left ventricular end-diastolic dimension; LVEF, left ventricular ejection fraction; NPPV, noninvasive positive pressure ventilation; NYHA, New York Heart Association.

**Table 2:** Laboratory data and medications used at discharge.

	Over-80	Under-80	
	*n* = 105	*n* = 75	*p*-Value
Laboratory data at discharge			
LVEF, %	51.9 ± 14.5	41.7 ± 17.2	0.0001
LVDd, mm	49.6 ± 7.6	54.9 ± 9.3	0.0002
Hemoglobin, g/dL	11.3 ± 2.0	13.3 ± 5.4	<0.0001
Total protein, g/dL	6.6 ± 0.6	6.6 ± 0.6	0.82
Albumin, g/dL	3.4 ± 0.4	3.5 ± 0.5	0.03
BUN, mg/dL	31.2 ± 15.6	23.5 ± 12.5	0.0003
Creatinine, mg/dL	1.3 ± 0.6	1.2 ± 0.6	0.11
eGFR, mL/min/1.73 m^2^	40.7 ± 17.7	53.4 ± 22.8	0.0001
Serum Na, mEq/L	138.7 ± 3.2	138.6 ± 3.0	0.92
Serum K, mEq/L	4.3 ± 0.5	4.4 ± 0.5	0.22
BNP, pg/mL	415.4 ± 545	418.1 ± 519	0.97
Medications at discharge			
ACEi/ARB	54 (51)	48 (64)	0.12
Beta blocker	70 (66)	66 (88)	0.001
Aldosterone blocker	51 (48)	48 (64)	0.04
Loop diuretics	91 (86)	49 (65)	0.001
Tolvaptan	64 (60)	43 (57)	0.64
Dose of tolvaptan, mg	6.6 ± 3.2	7.3 ± 3.2	0.35

Values are presented as the mean ± standard deviation or *n* (%). ACEi, angiotensin-converting enzyme inhibitor; ARB, angiotensin receptor blocker; BNP, brain natriuretic peptide; BUN, blood urea nitrogen; eGFR, estimated glomerular filtration rate; LVDd, left ventricular end-diastolic dimension; LVEF, left ventricular ejection fraction.

**Table 3:** Clinical events within one year of discharge.

	Over-80	Under-80	
	*n* = 88	*n* = 67	*p*-Value
MACCE	22 (25)	14 (20)	0.59
All-cause death	11 (12)	2 (2)	0.01
Cardiac death	7 (7)	1 (1)	0.13
Heart failure death	6 (6)	1 (1)	0.14
Noncardiac death	4 (4)	0 (0)	0.1
Rehospitalization due to heart failure	16 (18)	12 (17)	0.94
Cerebral infarction	0 (0)	1 (1)	0.43
Fatal arrhythmia	0 (0)	1 (1)	0.43

Categorical variables are presented as numbers (percentages). MACCE, major adverse cardiac and cerebrovascular events.

**Table 4:** Efficacy and safety of TLV, and in-hospital death during hospitalization.

	Over-80	Under-80	
	*n* = 109	*n* = 76	*p*-Value
In-hospital death, *n* (%)	4 (3)	1 (1)	0.65
Length of Hospitalization, days	19.2 ± 13.7	18.8 ± 8.1	0.8
Urine Volume			
0–24 h, mL	2620 ± 1227	3725 ± 2244	<0.001
0–48 h, mL	4854 ± 1969	6695 ± 3951	<0.001
Mean change in body weight, kg			
<7 days, kg	−4.8 ± 4.9	−6.7 ± 10.0	0.13
at discharge, kg	−5.9 ± 3.5	−7.2 ± 4.8	0.06
Hypernatremia			
<3 days, *n* (%)	3 (2)	4 (9)	0.44
In-hospital periods, *n* (%)	6 (5)	4 (9)	1
WRF, *n* (%)	13 (11)	6 (7)	0.46

Values are presented as the mean ± standard deviation or number (percentage). WRF, worsening renal failure; TLV, tolvaptan.

## Data Availability

The data that support the findings of this study are available from the corresponding author on reasonable request.

## References

[1] Sato N., Kajimoto K., Keida T., Mizuno M., Minami Y., Yumino D., Asai K., Murai K., Muanakata R., Aokage T. (2013). Clinical features and outcome in hospitalized heart failure in Japan (from the ATTEND Registry). Circ. J..

[2] Yasuda S., Nakao K., Nishimura K., Miyamoto Y., Sumita Y., Shishido T., Anzai T., Tsutsui H., Ito H., Komuro I. (2016). The current status of cardiovascular medicine in Japan -analysis of a large number of health records from a national-wide claim-based database, JROAD-DPC. Circ. J..

[3] Mizuno M., Kajimoto K., Sato N., Yumino D., Minami Y., Murai K., Munakata R., Asai K., Keida T., Sakata Y. (2016). Clinical profile, management, and mortality in very-elderly patients hospitalized with acute decompensated heart failure: An analysis from the ATTEND registry. Eur. J. Intern. Med..

[4] Ide T., Kaku H., Matsushima S., Tohyama T., Enzan N., Funakoshi K., Sumita Y., Nakai M., Nishimura K., Miyamoto Y. (2021). Clinical characteristics and outcomes of hospitalized patients with heart failure from the large-scale Japanese Registry of Acute Decompensated Heart Failure (JROADHF). Circ. J..

[5] Ushigome R., Sakata Y., Nochioka K., Miyata S., Miura M., Tadaki S., Yamauchi T., Sato K., Onose T., Tsuji K. (2015). Temporal trends in clinical cgaracteristics, management and prognosis of patients with symptomatic heart failure in Japan- report from the CHART studies. Circ. J..

[6] Mogensen U.M., Ersbøll M., Andersen M., Andersson C., Hassager C., Torp-Pedersen C., Gustafsson F., Køber L. (2011). Clinical characteristics and major comorbidities in heart failure patients more than 85 years of age compared with younger age groups. Eur. J. Heart Fail..

[7] Herrero-Puente P., Martín-Sánchez F.J., Fernández-Fernández M., Jacob J., Llorens P., Miró Ò., Alvarez A.B., Pérez-Durá M.J., Alonso H., Garrido M. (2012). Differential clinical characteristics and outcome predictors of acute heart failure in elderly patients. Int. J. Cardiol..

[8] Matsuzaki M., Hori M., Izumi T., Fukunami M., Tolvaptan Investigators (2011). Efficacy and safety of tolvaptan in heart failure patients with volume overload despite the standard treatment with conventional diuretics: A phase III, randomized, double-blind, placebo-controlled study (QUEST study). Cardiovasc. Drugs Ther..

[9] Konstam M.A., Gheorghiade M., Burnett J.C., Grinfeld L., Maggioni A.P., Swedberg K., Udelson J.E., Zannad F., Cook T., Ouyang J. (2007). Effects of oral tolvaptan in patients hospitalized for worsening heart failure: The Everest outcome trial. JAMA.

[10] Kinugawa K., Inomata T., Sato N., Yasuda M., Shimakawa T., Bando K., Mizuguchi K. (2015). Effectiveness and adverse events of tolvaptan in octogenarians with heart failure. Interim analyses of Samsca post-marketing surveillance in heart faiLurE (SMILE study). Int. Heart J..

[11] Niikura H., Iijima R., Anzai H., Kogame N., Fukui R., Takenaka H., Kobayashi N. (2017). Clinical utility of early use of tolvaptan in very elderly patients with acute decompensated heart failure. Anatol. J. Cardiol..

[12] Sato Y., Uzui H., Mukai M., Shiomi Y., Hasegawa K., Ikeda H., Tama N., Fukuoka Y., Morishita T., Ishida K. (2020). Efficacy and safety of tolvaptan in patients more than 90 years old with acute heart failure. J. Cardiovasc. Pharmacol. Ther..

[13] Morita Y., Endo A., Kagawa Y., Yamaguchi K., Sato H., Ouchi T., Watanabe N., Tanabe K. (2021). Clinical effectiveness and adverse events associated with tolvaptan in patients above 90 years of age with acute decompensated heart failure. Heart Vessels.

[14] McKee P.A., Castelli W.P., McNamara P.M., Kannel W.B. (1971). The natural history of congestive heart failure: The Framingham study. N. Engl. J. Med..

[15] Kimura K., Momose T., Hasegawa T., Morita T., Misawa T., Motoki H., Izawa A., Ikeda U. (2016). Early administration of tolvaptan preserves renal function in elderly patients with acute decompensated heart failure. J. Cardiol..

[16] Matsukawa R., Kubota T., Okabe M., Yamamoto Y., Meno H. (2018). Efficacy and safety of the early use of V2 receptor antagonists in elderly patients with decompensated heart failure. Heart Vessels.

[17] Kinoshita M., Okayama H., Kosaki T., Hosokawa S., Kawamura G., Shigematsu T., Takahashi T., Kawada Y., Hiasa G., Yamada T. (2018). Favorable effects of early tolvaptan administration in very elderly patients after repeat hospitalizations for acute decompensated heart failure. Heart Vessels.

[18] Katsanos S., Bistola V., Parissis J.T. (2015). Acute heart failure syndromes in the elderly: The European perspective. Heart Fail. Clin..

[19] Ejiri K., Noriyasu T., Nakamura K., Ito H. (2019). Unprecedented crisis-heart failure hospitalizations in current or future Japan. J. Cardiol..

[20] Matsukawa R., Kubota T., Okabe M., Yamamoto Y. (2016). Early use of V2 receptor antagonists is associated with a shorter hospital stay and reduction in in-hospital death in patients with decompensated heart failure. Heart Vessels.

[21] Shirakabe A., Asai K., Otsuka T., Kobayashi N., Okazaki H., Matsushita M., Shibata Y., Goda H., Shigihara S., Asano K. (2020). Clinical approach to shortening length of hospital stay in elderly patients with acute heart failure requiring intensive care. Circ. Rep..

[22] Kiuchi S., Fujii T., Hisatake S., Kabuki T., Takashi O., Dobashi S., Ikeda T. (2017). Experience with long-term administration of tolvaptan to patients with acute decompensated heart failure. Drug Discov. Ther..

[23] Nishino M., Tanaka A., Kawanami S., Sugae H., Ukita K., Kawamura A., Nakamura H., Matsuhiro Y., Yasumoto K., Tsuda M. (2022). Suitable dose of long-term tolvaptan to reduce heart failure rehospitalizations. Int. Heart J..

[24] Jessup M., Abraham W.T., Casey D.E., Feldman A.M., Francis G.S., Ganiats T.G., Konstam M.A., Mancini D.M., Rahko P.S., Silver M.A. (2009). 2009 focused update: ACCF/AHA guidelines for the diagnosis and management of heart failure in adults: A report of the American College of Cardiology Foundation/American Heart Association Task Force on Practice Guidelines: Developed in collaboration with the International Society for Heart and Lung Transplantation. Circulation.

[25] Ambrosy A.P., Pang P.S., Khan S., Konstam M.A., Fonarow G.C., Traver B., Maggioni A.P., Cook T., Swedberg K., Burnett J.C. (2013). Clinical course and predictive value of congestion during hospitalization in patients admitted for worsening signs and symptoms of heart failure with reduced ejection fraction: Findings from the Everest trial. Eur. Heart J..

[26] Tromp J., Ouwerkerk W., van Veldhuisen D.J., Hillege H.L., Richards A.M., van der Meer P., Anand I.S., Lam C.S.P., Voors A.A. (2022). A systematic review and network meta-analysis of pharmacological treatment of heart failure with reduced ejection fraction. JACC Heart Fail..

[27] Jorge R.-G., Biniyam G.D., Jozine M.M., John G.C., Christopher M.O., Macro M., Piotr P., John R.T., Gad C., Beth A.D. (2018). Prevalence, predictors and clinical outcome of residual congestion in acute decompensated heart failure. Int. J. Cardiol..

[28] Sofia I.L., Luis R.D.S., Ines A., Filipa M., Patricia B., Augusta G., Candida F. (2018). A 2018 overview of diuretic resistance in heart failure. Rev. Port. Cardiol..

[29] Ida L., Karolina S., Marie E., Juan-Jesus C., Lars H.L., Tomas J. (2019). Incidence of, associations with and prognostic impact of worsening renal function in heart failure with different ejection fraction categories. Am. J. Cardiol..

[30] Salah K., Kok W.E., Eurlings L.W., Bettencourt P., Pimenta J.M., Metra M., Verdiani V., Tijssen J.G., Pinto Y.M. (2015). Competing risk of cardiac status and renal function during hospitalization for acute decompensated heart failure. JACC Heart Fail..

[31] Raja C., Ranjeeta C., David S.G. (2022). Hypernatremia in the intensive care unit. Curr. Opin. Nephrol. Hypertens..

[32] Zhili Q., Jiaqi L., Pei L., Tian L., Ang L., Meili D. (2023). Monogram prediction model of hypernatremia on mortality in critically III patients. Infect. Drug Resist..

